# A Well-Resolved Phylogeny of the Trees of Puerto Rico Based on DNA Barcode Sequence Data

**DOI:** 10.1371/journal.pone.0112843

**Published:** 2014-11-11

**Authors:** Robert Muscarella, María Uriarte, David L. Erickson, Nathan G. Swenson, Jess K. Zimmerman, W. John Kress

**Affiliations:** 1 Department of Ecology, Evolution and Environmental Biology, Columbia University, New York, New York 10027, United States of America; 2 Department of Botany, MRC-166, National Museum of Natural History Smithsonian Institution, P.O. Box 37012, Washington, D. C., 20013, United States of America; 3 Department of Plant Biology, Michigan State University, East Lansing, Michigan 48824, United States of America; 4 Department of Environmental Science, University of Puerto Rico, San Juan, Puerto Rico 00925, United States of America; The New York Botanical Garden, United States of America

## Abstract

**Background:**

The use of phylogenetic information in community ecology and conservation has grown in recent years. Two key issues for community phylogenetics studies, however, are (i) low terminal phylogenetic resolution and (ii) arbitrarily defined species pools.

**Methodology/principal findings:**

We used three DNA barcodes (plastid DNA regions *rbcL*, *matK*, and *trnH-psbA*) to infer a phylogeny for 527 native and naturalized trees of Puerto Rico, representing the vast majority of the entire tree flora of the island (89%). We used a maximum likelihood (ML) approach with and without a constraint tree that enforced monophyly of recognized plant orders. Based on 50% consensus trees, the ML analyses improved phylogenetic resolution relative to a comparable phylogeny generated with Phylomatic (proportion of internal nodes resolved: constrained ML = 74%, unconstrained ML = 68%, Phylomatic = 52%). We quantified the phylogenetic composition of 15 protected forests in Puerto Rico using the constrained ML and Phylomatic phylogenies. We found some evidence that tree communities in areas of high water stress were relatively phylogenetically clustered. Reducing the scale at which the species pool was defined (from island to soil types) changed some of our results depending on which phylogeny (ML vs. Phylomatic) was used. Overall, the increased terminal resolution provided by the ML phylogeny revealed additional patterns that were not observed with a less-resolved phylogeny.

**Conclusions/significance:**

With the DNA barcode phylogeny presented here (based on an island-wide species pool), we show that a more fully resolved phylogeny increases power to detect nonrandom patterns of community composition in several Puerto Rican tree communities. Especially if combined with additional information on species functional traits and geographic distributions, this phylogeny will (i) facilitate stronger inferences about the role of historical processes in governing the assembly and composition of Puerto Rican forests, (ii) provide insight into Caribbean biogeography, and (iii) aid in incorporating evolutionary history into conservation planning.

## Introduction

The use of phylogenetic information in community ecology and conservation has grown dramatically in recent years [1], [2], [3]. This body of research has been largely stimulated by the idea that evolutionary relationships can provide insights into the historical processes governing assembly of local communities [4], [5], [6]. From a conservation perspective, phylogenies may reveal aspects of biodiversity that are not observable from traditional metrics of species diversity [7], [8], [9], . By providing a historical context, phylogenies help merge our understanding of ecological, evolutionary, and biogeographic drivers of community composition [12].

One key issue for research in community phylogenetics is how to best estimate phylogenetic relationships among species in diverse communities (*e.g.*, tropical forests). To date, the program Phylomatic [13] has become a primary method by which ecologists integrate phylogenetic information with analyses of community patterns (*e.g.,* [14], [15], [16]). For plants, Phylomatic generates community phylogenies by pruning a megatree of angiosperms given a user-defined species list. This approach offers a repeatable and accessible way to obtain phylogenies using existing data (*also see* [17]), however, Phylomatic phylogenies typically have low or no taxonomic resolution among closely related species (*e.g.*, within plant families or genera). Low taxonomic resolution can reduce statistical power for detecting nonrandom patterns of community structure [18], [19] and can bias estimates of phylogenetic signal [20]. Furthermore, because single genera often contain numerous species with diverse life-history characteristics (*e.g.,* [21], [22]), resolving evolutionary relationships among congeners is critical for interpreting the link between patterns of phylogenetic community composition and the history of trait evolution. Finally, low taxonomic resolution can preclude inferences about biogeographic influences on local assemblages. The issue is particularly acute with respect to relatively recent evolutionary history (i.e., speciation events), which arguably represent a key connection between local and regional processes (*see* [23] *and references therein*).

In contrast to megatree approaches such as Phylomatic, phylogenies based on genetic data typically provide comparatively high taxonomic resolution. Generating molecular phylogenies, however, requires a significant investment of resources and expert knowledge. Additionally, determining how to estimate phylogenies among the very distantly related species that are typical of community-based phylogenies (as opposed to clade-based phylogenies) remains an active area of research. One potentially promising approach is to integrate existing information on evolutionary relationships in the form of a constraint tree [24]. More research is required, however, to determine the influence of constraint trees on phylogenetic reconstruction and downstream analyses of community phylogenetic patterns.

Another characteristic of many existing studies of community phylogenetic structure lies in the lack of consistent methodology in defining species pools when testing hypotheses about mechanisms driving community assembly (*e.g.*, competition versus environmental filtering) [4]. Generally, these analyses are based on null models that compare an observed metric of phylogenetic composition (*e.g.*, NRI­, the net relatedness index) with a random expectation based on assemblages drawn from a regional species pool [16]. In practice, studies often delimit the ‘regional pool’ as the set of species encountered in the study, regardless of the ecological significance of the study area boundaries (*e.g.*, forest dynamics plots). Examining species assemblages within such arbitrarily defined regions can provide information on processes occurring at certain scales (*e.g.,* [16], [25]). However, varying the spatial scale at which species pools are defined can provide important opportunities to evaluate the relative strength of local assembly processes (*e.g.*, interactions that occur among neighboring trees) versus processes that occur over larger spatial and temporal scales (*e.g.*, evolution and biogeography) and across broader environmental gradients (*e.g.,* [26], [27], [28], [29], [30], [31]). For example, numerous studies in phylogenetic community ecology have shown that as the spatial (and taxonomic) extent of the species pool increases, the phylogenetic composition of local communities tends to appear increasingly ‘clustered’ (*i.e.*, co-occurring species are more closely related than expected by random chance). Other studies have shown more mixed results (*see references in* [31], [32]), which may emerge, for example, if a larger species pool includes sister taxa absent from the smaller pool. In any case, scale-dependency of community patterns likely reflects the scales at which different assembly processes influence community structure [5], [33], [34], [35]. As such, we can gain valuable insights on community assembly by adjusting species pools to suit particular hypotheses about the scales at which different assembly processes act [6], [28], [29], [31], [36], [37], [38], [39].

In this study, we used DNA sequence data to generate an island-wide phylogeny for nearly all of the native and naturalized tree species of Puerto Rico. Specifically, we used sequence data from three regions of plastid DNA which are commonly used as plant DNA barcodes (*rbcL*, *matK*, *trnH*-*psbA*; [24]) to resolve evolutionary relationships among 527 recognized species with a maximum likelihood (ML) approach. We compare phylogenetic resolution of two ML phylogenies (built with and without the use of an ordinal-level constraint tree) and a comparable phylogeny derived from Phylomatic. We then explore the implications of these different methods in a case study where we examined the phylogenetic structure of tree communities in 15 protected forests in Puerto Rico. These 15 forests span a wide variation in environmental conditions, providing an ideal template for evaluating the effects of local environmental variation on phylogenetic community structure within the island of Puerto Rico (Table 1). We addressed the following specific questions:

**Table 1:** Environmental characteristics and generalized results of ‘nodesig’ analysis (*i.e.,* over and underrepresented lineages) for 15 protected forests in Puerto Rico.

Forest	Area (ha)^1^	Holdridge Life Zone(s)^2^	Elevation Range (mean) (m)^3^	Mean Annual Precipitation (mm yr^−1^)^4^	Primary Geologic Substrate^5^	Species Richness^6,7^	Overrepresented Groups	Underrepresented Groups
Aguirre	432	df-S	0–4 (1)	953	Unconsolidated	33	Combretaceae, Fabaceae	
Boquerón	623	df-S	0–5 (1)	786	Unconsolidated	19	Combretaceae	
Cambalache	649	mf-S	31–188 (157)	1,593	Limestone	152	Arecaceae, Burseraceae, Anacardiaceae, Celastraceae	Melastomataceae
Carite	2,699	wf-S, wf-LM	296–839 (657)	2,018	Volcanic	146	Lauraceae, Solanaceae, *Psychotria* (Rubiaceae), *Myrcia* (Myrtaceae), *Clusia* (Clusiaceae), Meliaceae	*Exostema, Guettarda*, and *Stenostomum* (Rubiaceae), Fabaceae (Mimosoidae)
Ceiba	237	df-S	0–11 (4)	1,408	Unconsolidated	5	Solanaceae, Combretaceae, Rhizophoraceae	
Guajataca	955	mf-S	192–310 (249)	1,981	Limestone	197	Nyctaginaceae, Sapindales, Meliaceae	
Guánica	3,831	df-S	0–210 (81)	876	Limestone	133	*Coccoloba* (Polygonaceae), *Crescentia* (Bignoniaceae), Capparaceae, Sapindales, Fabales	Melastomataceae, Laurales, Ericales
Monte Guilarte	1,705	wf-S, wf-LM	629–1079 (909)	2,156	Volcanic	87	*Piper* (Piperaceae), *Miconia* (Melastomataceae), Meliaceae, *Inga* (Fabaceae)	
El Yunque	11,429	mf-S, wf-S, wf-LM, rf-S, rf-LM	87–1011 (570)	3,758	Volcanic	215	Solanaceae, Melastomataceae, Meliaceae, Laurales	Rutaceae, Fabaceae, Celastraceae
Maricao	4,168	mf-S, wf-S, wf-LM	130–871 (511)	2,126	Serpentine	212	Araliaceae, Aquifoliaceae, Meliaceae	Lamiales
Rio Abajo	2,284	mf-S, wf-S	209–380 (313)	2,079	Limestone	175	Meliaceae	
Piñones	732	mf-S	0–2 (1)	1,398	Unconsolidated	31	Combretaceae, Malvaceae, *Pterocarpus* (Fabaceae)	
Susúa	1,298	mf-S	107–501 (264)	1,395	Serpentine	180	Rubiaceae	Melastomataceae
Toro Negro	2,763	wf-S, wf-LM	486–1284 (988)	2,248	Volcanic	133	Aquifoliaceae, Primulaceae, Meliaceae, *Piper* (Piperaceae), Araliaceae, Laurales, Solanaceae, Melastomataceae	*Guettarda* and *Stenostomum* (Rubiaceae), Fabaceae
Vega	482	mf-S	27–110 (67)	1,668	Limestone	86	Arecaceae, Meliaceae, Celastraceae, Moraceae, Urticaceae *Drypetes* (Putrajivaceae)	

Environmental and occurrence data are from ^1^Gould *et al.* [90], ^2^Ewel & Whitmore [41], ^3^Gesch [91], ^4^Daly *et al*. [42], ^5^ [43], ^6^Little & Wadsworth [63], and ^7^Little *et al*. [64]. Forest life zones are coded as: subtropical dry (df-S), subtropical moist (mf-S), subtropical wet (wf-S), lower montane wet (wf-LM), subtropical rainforest (rf-S), lower montane rainforest (rf-LM).

1. How does the use of a constraint tree influence (i) the level of bootstrap support in a DNA barcode phylogeny of Puerto Rican trees, and (ii) the degree to which a molecular phylogeny corresponds with currently recognized taxonomic groups? We predicted that the constraint tree would provide higher levels of bootstrap support among unconstrained nodes and increase concordance with current taxonomy relative to the unconstrained analysis.
2. How do patterns of community phylogenetic structure in Puerto Rican forests differ when based on a DNA barcode phylogeny versus a Phylomatic phylogeny? We predicted that an increase in statistical power provided by the higher resolution of a molecular phylogeny would lead to a stronger signal of non-random phylogenetic structure.
3. How does phylogenetic structure in Puerto Rican forests change with respect to different species pool definitions? We predicted co-occurring species would tend to appear relatively phylogenetically clustered with respect to the full island species pool because of a strong role for environmental filtering across broad environmental gradients. We predicted that a more restricted species pool definition would reduce the level of phylogenetic clustering if niche differentiation (competitive exclusion) becomes more apparent at small spatial scales.

## Materials and Methods

All necessary permits were obtained for the described study, which complied with all relevant regulations. Specifically, the Departmento de Recursos Naturales y Ambientales (DRNA) of Puerto Rico granted permit #2011-IC-046 to collect plant specimens in the state forests of Puerto Rico. Herbaria staff at the University of Puerto Rico, Rio Piedras and the US National Herbarium provided permission to sample tissue from their collections.

### Study area and species

The island of Puerto Rico encompasses six Holdridge life zones [40] ranging from subtropical dry forest to subtropical rainforest in an area of 8,740 km^2^ [41]. Mean annual precipitation ranges drastically, from ca. 700–4,500 mm yr^−1^ [42]. The island’s complex geologic history is reflected in its rugged topography (0–1,338 m a.s.l.) and diverse parent soil materials, which include volcanic, limestone, alluvial, and ultramafic materials [43]. Substantial existing data on the flora (*e.g.,* [24], [44], [45]) provide a strong foundation for our work.

We created an initial list of Puerto Rican trees with the species list from the USFS Forest Inventory and Analysis (FIA) Caribbean field guide [46]. With guidance from local experts (P. Acevedo-Rodríguez, F. Areces, F. Axelrod, M. Caraballo, J. Sustache, and P. Vives, *personal communication*), we modified this list by (1) updating nomenclature to be consistent with Acevedo-Rodríguez and Strong [45], (2) removing species occurring only under cultivation and (3) adding native and naturalized tree species known to occur in Puerto Rico but absent from the FIA list. Our final list of target species contained 594 species of seed plants representing 33 orders, 86 families, and 304 genera (Table S1). Of these, we were able to compile DNA sequence data for 523 (89%) species representing all 32 orders, 85 families (99%), and 287 genera (94%). The single excluded family (Cunoniaceae) is represented in Puerto Rico by a single rare species of shrub and most of the other species missing from our dataset are relatively uncommon and distributed widely throughout taxonomic groups. As a result, we do not expect the missing species to influence overall results of community phylogenetic analyses. However, it will be enlightening to include these species when sequence data become available in order to better understand the contributions of rare species to phylogenetic diversity [47].

### Tissue collection and lab procedures

We acquired DNA sequence data from a variety of sources. Primarily, we obtained leaf tissue either from freshly collected specimens or existing herbarium sheets. For fresh specimens, we dried leaf tissue in silica gel prior to DNA extraction. Prior to depositing voucher specimens at the US National Herbarium (US), we verified species identifications by referring to the herbarium at the University of Puerto Rico, Río Piedras (UPRRP) and through consultation with local experts (F. Areces, F. Axelrod, P. Vives, *personal communication*). For 95 species, we collected leaf tissue from dry material sampled from herbarium specimens at UPRRP or US. DNA extraction, amplification and sequencing protocols followed Kress *et al*. [24]. Specifically, we used the following lab procedures for fresh and dried leaf tissue. After disrupting tissue with a Tissuelyzer (Qiagen Cat. #85210), we incubated samples overnight at 55°C in a CTAB-based extraction buffer (AutoGen, Holliston, MA). Following incubation, we removed the supernatant and placed it in clean, 2 ml 96-well plate for submission to a DNA extraction robot (AutoGen 960, Holliston, MA). We hydrated DNA extractions in 100 mM Tris-HCl (pH 8.0) and then transferred them to Matrix barcode tubes (MatrixTechnologies Cat. # 3735) and stored them at −80°C. Working stocks of DNA were transferred to a microtiter plate and diluted 5× with water prior to PCR. We used routine PCR, with no more than three attempts per sample to recover PCR amplicons for each sample. The PCR cycling conditions were exactly the same for *rbcL* and *trnH-psbA* (95°C 3 min, [94°C 30 sec, 55°C 30 sec, 72°C 1 min]×35 cycles, 72°C 10 min) following procedures outlined in Kress and Erickson [48]. The PCR cycling conditions for *matK* required lower annealing temperatures and more cycles (95°C 3 min [94°C 30 sec, 49°C 30 sec, 72°C 1 min]×40 cycles, 72°C 10 min) following Fazekas *et al*. [49] and included DMSO at a final concentration of 5%. We purified successful PCR reactions with a 56 diluted mixture of ExoSap (USB, Cat. # 78201). For sequencing, 2–4 ul of the purified PCR was used in a 12 ul reaction (0.8 ul BigDye terminator sequencing mixture (V3.1; ABI, Cat. 4337457), 2.0 ul of a 56 buffer (400 u Molar Tris-HCL pH 8.0), 1 ul of 1 uMolar primer and distilled water to volume). Sequencing of *matK* PCR products included DMSO to a final concentration of 4% in the reaction mixture. Cycling sequencing protocols were the same for all markers, (95°C 15 sec [94°C 15 sec, 50°C 15 sec, 60°C 4 min]×30 cycles). Following cycle sequencing, products were purified on a column of sephadex and sequence reactions were read on an ABI 3730 (Applied Biosystems).

We also incorporated existing sequence data for 143 species previously sequenced from the Luquillo Forest Dynamics Plot [24] and for 25 species from GenBank [50]. We excluded 67 species from analyses for which we were unable to acquire reliable sequence data either because tissue was not available or because of failure during DNA sequencing (Table S1).

### Sequence editing, alignment, and assembly

We used Geneious (R6, version 2.4.1; Biomatters Ltd.) to trim and assemble trace files for each marker into bidirectional contigs. Separately for each marker, we aligned sequences using SATé [51]. SATé is an iterative algorithm that divides the original sequence data set using a tree-based decomposition; we aligned these smaller sets of sequences using MAFFT [52] and merged these sub-alignments into a global alignment without disrupting the individual sub-alignments using MUSCLE [53]. SATé is particularly effective for conducting multiple sequence alignment among very distantly related taxa through the use of merging sub-alignments among related sequences, and has been widely applied for studies of very broad phylogenetic application [54], [55]. We then concatenated the three separate marker alignments to produce an aligned three-gene matrix. Gaps were not coded and were treated as missing data in phylogenetic reconstruction.

### Phylogenetic reconstruction

We generated a phylogeny using maximum likelihood (ML) methods, implemented in RAxML (Stamatakis et al. 2005) via the CIPRES Science Gateway [56]. Based on jModelTest2 [57], we modeled nucleotide substitution using a GTR+GAMMA model, with substitution rates estimated independently for each gene. We evaluated node support for the topology with the highest likelihood using 100 bootstrap runs. In addition, we trimmed Phylomatic reference tree R20120829 [58] to use for comparative purposes. While other methods for phylogenetic reconstruction are available (*e.g.*, parsimony), we focus here on a comparison between ML methods and a very commonly used method of generating phylogenies for community ecology (Phylomatic).

Rather than including densely sampled small taxonomic units, community phylogenies often contain smaller numbers of more distantly related species (*e.g.*, 32 orders represented in our dataset, represented by 18 species, on average). Resolving both shallow and deep relationships requires distinct molecular data sets that are difficult to assemble. When strong prior information is available, one approach to confront this issue is to enforce some relationships through the use of a constraint tree (*see for example* [59]). In the case of our study, the Angiosperm Phylogeny Group III [60] represents the authoritative standard for current relationships up to the family level in angiosperms. However, within the AGP III phylogeny, relationships between species are generally not resolved beyond the family level, thus providing an ideal opportunity to use DNA barcodes to resolve these finer-scale relationships. To test the ability of a constraint tree to improve phylogenetic resolution among distantly related taxa, we repeated the ML analysis detailed above using the APG III phylogeny [60] to constrain the topology of ordinal and deeper nodes. This approach allowed the topology within each order to be resolved with DNA barcode sequence data while ordinal and deeper nodes were enforced *a priori*. We dated both the constrained and unconstrained ML phylogenies using PATHd8 [61] with age constraints based on fossil records provided in the Appendix of Magallón & Castillo [62] (input files for our analyses are provided in Appendix S1 and S2). The constraints we used included one fixed age estimate for the angiosperm crown group and 35 minimal age estimates for other clades represented in our phylogeny ([62]; Appendix S1 and S2). We used this approach because dated ultrametric trees are the standard for community phylogenetics studies; however, we also provide the undated, non-ultrametric trees in Appendix S3. To explore the distribution of uncertainty across the phylogeny, we calculated the proportion of recognized taxonomic groups (orders, families, and genera) that were found to be monophyletic in each analysis and the proportion of resolved nodes within each of these groups.

### Case study: Phylogenetic composition of Puerto Rican forests

**Figure 1:**
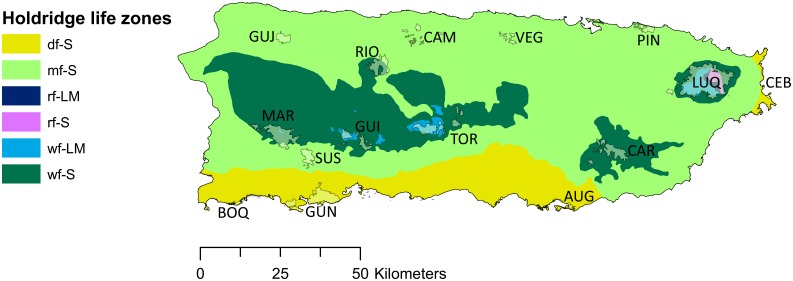
A map of Puerto Rico including the 15 state forests used in this study [90]. Forest life zones are coded as: subtropical dry (df-S), subtropical moist (mf-S), subtropical wet (wf-S), lower montane wet (wf-LM), subtropical rainforest (rf-S), lower montane rainforest (rf-LM). Refer to Table 1 for forest codes.

We measured the phylogenetic composition of 15 protected forests in Puerto Rico based on species occurrence data (presence/absence) from Little & Wadsworth [63] and Little *et al*. [64]. As a synthesis of observations made by local experts, these volumes are the most commonly used references to describe tree composition of Puerto Rico’s protected forests. The 15 forests examined here span a wide range of environmental conditions (precipitation range: ca. 800–3,800 mm yr^−1^, elevation range: ca. 0–1,300 m a.s.l.) and occur across four main soil parent materials: unconsolidated, limestone, volcanic, and serpentine (Table 1, Fig. 1). We excluded taxa not included in our phylogeny – these accounted for only 2% of the total observations in the community dataset. With the remaining data, we quantified phylogenetic composition of each forest using the net relatedness index (NRI) and nearest taxon index (NTI) [4]. These indices describe whether sets of co-occurring taxa are more or less closely related than random assemblages of equal species richness drawn from a pool of species. Specifically, NRI measures the average degree of relatedness among all members of the community and thus emphasizes deeper branches of the phylogenetic tree. In contrast, NTI is based on the average distance between closest relatives in each assemblage and thus emphasizes compositional patterns at the tips of the phylogeny [4]. These metrics are calculated as: NRI = –(*r*_obs_–mean(*r*_rand_))/sd(*r*_rand_), where *r* is either the co-occurring taxa (for NRI) or mean phylogenetic branch length separating nearest neighbors (for NTI). The observed value is *r*_obs_ and *r*_rand_ is a distribution of values based on assemblages drawn from a species pool. We calculated NRI and NTI for each forest using two different species pools: the full list of species in our dataset (the ‘island pool’), and the list of species recorded from forests on the same soil parent material (the ‘soil pool’). For example, for Guánica forest (limestone soil), we calculated two values of NRI: one value (NRI_ISLAND_) based on null assemblages drawn from the entire species list and another value (NRI_SOIL_) based on the list of species recorded from all forests on limestone soil (the soil pool).

We computed NRI and NTI using the ses.mpd and ses.mntd functions of the ‘picante’ package [65] for R v 3.0.0 [66]. We ran the analyses for 999 iterations and used the ‘taxa-labels’ null model. We chose this null model to control for the observed species occupancy rates and species richness of each forest. Positive values of NRI and NTI indicate phylogenetic clustering whereas negative values indicate phylogenetic evenness. We performed these analyses using the constrained ML 50% consensus tree and the Phylomatic phylogeny. We based these analyses on the constrained ML 50% consensus tree because it reflects the uncertainty of our phylogenetic hypothesis given our data, while also incorporating the strong evidence resolving deep relationships provided by the APG III constraint tree.

We quantified shifts in NRI and NTI values between the two species pool definitions using paired t-tests and we quantified the similarity of these values between phylogenies with Pearson’s correlation coefficient. In addition to overall patterns of community phylogenetic composition, we used the ‘nodesig’ algorithm in Phylocom v 4.2 [67] to determine the particular clades that contribute significantly more or fewer species than expected to the composition of each forest.

## Results

### DNA barcode sequences

From fresh tissue, we successfully recovered sequence data from 85%, 75%, and 94% of samples for *rbcL*, *matK, trnH-psbA*, respectively. The final three-gene alignment comprised 3,366 base pairs (549 bp for *rbcL*, 1,070 bp for *matK*, and 1,747 bp for *trnH-psbA*). The data matrix had 62.2% missing data (including gaps coded as missing data and species for which we did not recover sequence data). This amount is far more compact than previous alignments of the same three regions that used a nested partitioning of the *trnH-psbA* alignment, resulting in >95% missing data [24]. Considering each region separately, the amount of missing data was 23.1%, 49.2%, and 82.1% for *rbcL, matK,* and *trnH-psbA*, respectively.

### Phylogenetic analyses

**Figure 2:**
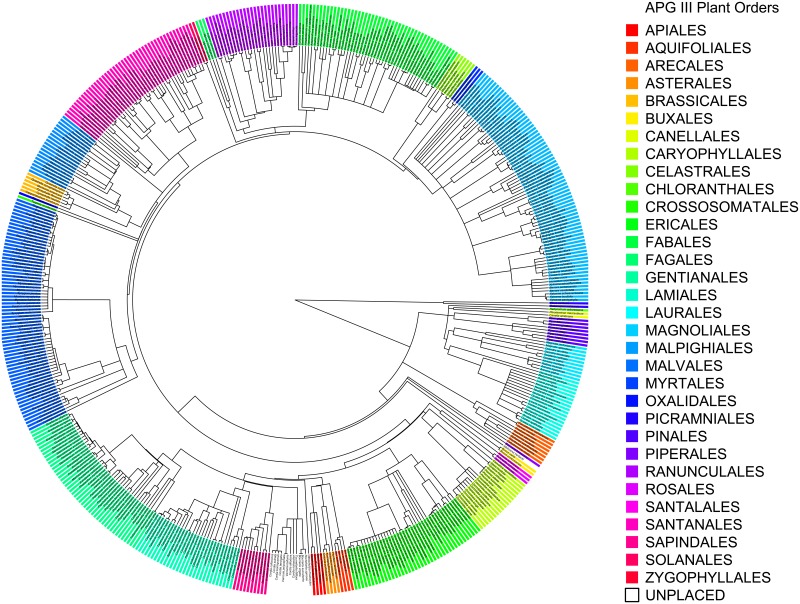
A maximum likelihood phylogeny constrained at the ordinal level representing 526 native and naturalized tree species of Puerto Rico (the single tree fern in the phylogeny is excluded to aid visualization). Ordinal placement according to APG III [60] is color coded.

We provide the constrained and unconstrained ML trees, with bootstrap support, as well as the Phylomatic phylogeny used in our analyses in Appendix S3. Overall, we found relatively strong support for the majority of nodes in the both the constrained and unconstrained ML trees (Fig. 2). Across all nodes, 74% of nodes in the constrained ML tree received ≥50% bootstrap support and 52% received ≥80% bootstrap support. Considering only the 468 unconstrained nodes, 71% received ≥50% bootstrap support and 46% received ≥80% bootstrap support. The unconstrained ML tree had slightly lower levels of support with 68% of nodes receiving ≥50% support and 43% of nodes receiving ≥80% support. Both the constrained and unconstrained ML trees had higher resolution than the Phylomatic tree, in which only 52% of internal nodes were resolved. For the constrained ML tree, monophyly was supported for 91% of families and 87% of genera (monophyly of orders was constrained). In comparison, monophyly was supported for 72% of orders, 85% of families, and 87% of genera in the unconstrained ML tree. In both cases, the non-monophyly of currently recognized families related to the placement of taxa for which we did not have sequence data for all three barcode regions. For the constrained ML tree, the average proportion of nodes within orders, families, and genera with ≥50% bootstrap support was 0.81 (± SD 0.20), 0.87 (± SD 0.20), and 94% (± SD 0.19), respectively. For the unconstrained ML tree, the average proportion of nodes within orders, families, and genera with ≥50% bootstrap support was 0.92 (± SD 0.14), 0.89 (± SD 0.18), and 92% (± SD 0.20), respectively.

### Case study: Phylogenetic composition of Puerto Rican forests

**Figure 3:**
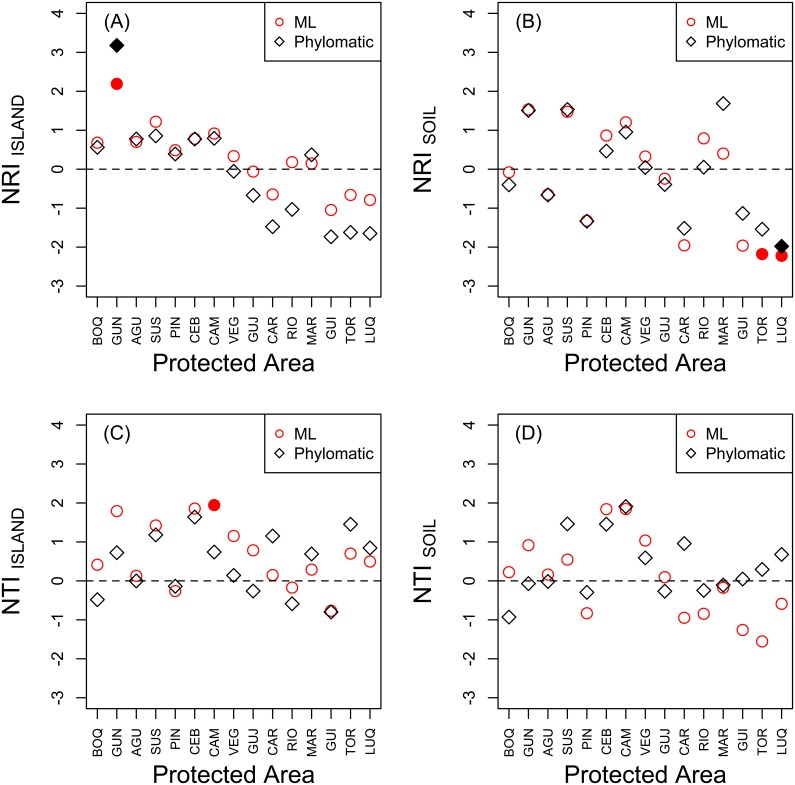
The net relatedness index (NRI) (A, B) and nearest taxon index (NTI; C, D) based on species occurrence records from Little & Wadworth [63] and Little *et al*. [64] versus reserve area [90] for 15 state forests in Puerto Rico. Leftmost panels are based on a null model using the full island species pool; right panels (B, D) are based on species pools restricted to primary soil types. Forests are sorted from left to right in order of their mean annual precipitation. Positive values indicate phylogenetic clustering and negative values indicate phylogenetic evenness. Filled symbols indicate values that are significantly different from a null model. Refer to Table 1 for forest codes.

Some patterns of phylogenetic community structure varied with respect to the phylogeny and species pool used in analyses (Fig. 3). For NRI, which emphasizes tree-wide patterns, Guánica dry forest was significantly clustered (i.e., taxa were more closely related than expected) based on the full island species pool for both the ML and Phylomatic phylogenies (Fig. 3A). None of the other 14 forests departed from random expectations for NRI when based on the island pool. When considering the (reduced) soil species pools, the composition of the two wettest forests (Toro Negro and El Yunque, both located on volcanic soils) were significantly overdispersed (i.e., taxa were less closely related than expected), although the NRI_SOIL_ value for Toro Negro was only significant with respect to the ML phylogeny (Fig. 3B). For NTI, which emphasizes compositional patterns at the tips of the phylogeny, Cambalache forest was significantly clustered with respect to the full island species pool but only for the ML phylogeny (Fig. 3C). None of the forests had significantly nonrandom NTI values when the analyses were based on the (reduced) soil species pools, regardless of which phylogeny was used (Fig. 3D).

None of the forests shifted from significantly clustered to significantly even when comparing NRI or NTI values based on the two different species pools. However, as we predicted, the (reduced) soil species pools caused both of these metrics to become more negative (i.e., decreased the signal of phylogenetic clustering) when calculated with the ML phylogeny (paired t-test: NRI: t = 2.79, df = 14, p<0.01; NTI: t = 4.34, df = 14, p<0.001). In contrast, these species pool definitions did not significantly change NRI or NTI when calculated with the Phylomatic phylogeny (paired t-test: NRI: t = 0.39, df = 14, p = 0.35; NTI: t = 0.28, df = 14, p = 0.39). Values of NRI calculated with each phylogeny were strongly correlated for both species pool definitions (island pool: Pearson’s *r* = 0.96, p<0.001; soil pool: Pearson’s *r* = 0.92, p<0.001) but values of NTI were less strongly correlated between these two phylogenies, and were not significantly correlated when based on the soil species pool (island pool: Pearson’s *r* = 0.60, p = 0.02; soil pool: Pearson’s *r* = 0.48, p = 0.06).

The node-based analysis identified particular clades that were relatively over- and under represented in each forest compared with a random expectation (Table 1) and, overall, the ML and Phylomatic phylogenies produced largely congruent results (Appendix S4). One of the more consistent results was that species belonging to Melastomataceae tended to be significantly underrepresented in relatively dry forests on limestone and serpentine soils (*i.e.*, Guánica, Cambalache, Maricao, and Susúa) and relatively overrepresented in three relatively wet forests on volcanic soils (Guilarte, Luquillo and Toro Negro). Also, phylogenetic clustering of Guánica forest appears to be primarily driven by an overrepresentation of Fabaceae and Capparaceae, together with an underrepresentation of magnoliids, Ericales, and Melastomataceae (Appendix S4).

## Discussion

The island-wide phylogeny for Puerto Rican trees presented here represents the community phylogenetics approach applied at a regional scale with the use of DNA sequence data. Both the constrained and unconstrained ML phylogenies provided increased phylogenetic resolution in comparison with a corresponding Phylomatic tree, a predominant tree-building approach used in studies of community phylogenetics. In this study, the use of an ordinal-level constraint tree provided slightly higher phylogenetic resolution compared to the unconstrained analysis. In our case study, we uncovered patterns of nonrandom phylogenetic structure in Puerto Rican forests that depended on the phylogeny used as well as the scale at which the regional species pool was defined. Considering the rapidly increasing availability of DNA sequence data, future regional scale work in community phylogenetics will benefit from highly resolved phylogenies that include many taxa sampled across large areas and broad environmental gradients [39], [68], [69].

### Comparison between phylogenies and taxonomic resolution

Although the ML phylogenies generated in this study were not completely resolved, the constrained 50% consensus tree did increase tip resolution by 22% in comparison with the Phylomatic tree. This relatively high degree of phylogenetic resolution has a number of important implications for community phylogenetic analyses [18], [19], [20]. First, poorly resolved phylogenies tend to reduce statistical power for detecting nonrandom patterns of community structure (*e.g.*, with NRI and NTI), an issue that appears to be more severe with larger phylogenies [18]. Swenson [18] found that statistical power was most strongly reduced, however, when deeper nodes were unresolved (i.e. among orders and families) as opposed to more recent nodes (i.e. among species). As a result, we expect that the remaining unresolved nodes in our ML tree have a relatively small effect on analyses of phylogenetic structure for Puerto Rican tree communities because our constraint tree fixed the resolution of the deeper nodes. At the same time, the relatively deep nodes of the Phylomatic phylogeny are also resolved, suggesting that a reduction in statistical power for detecting nonrandom patterns between our ML tree and the Phylomatic tree may be most pronounced for metrics that focus on phylogenetic patterns among close relatives (*e.g.*, NTI).

A second issue related to poorly resolved phylogenies is an upward bias when estimating phylogenetic signal [20]. In other words, the tendency for close relatives to have similar functional traits tends to be overestimated when phylogenies are poorly resolved. This bias is of particular concern when examining patterns of phylogenetic community composition given the central role of phylogenetic signal of traits relevant for species co-occurrence [70]. In general, the relatively high degree of tip resolution afforded by molecular data can strengthen inferences that rely on linking phylogenetic and functional patterns of community composition.

A major challenge in generating large-scale community phylogenies (and systematic biology, in general) is how to recover accurate phylogenetic relationships given limited data. Researchers have long debated the relative benefits of increasing sequence length versus increasing taxon sampling to improve the accuracy of phylogenetic reconstruction (*e.g.,* [71], [72], [73], [74], [75]). This issue, however, has rarely been discussed in the context of community phylogenetics even though community-based analyses typically have relatively sparse taxon sampling compared to clade-based analyses. One implication of sparse taxon sampling is that long-branch attraction can reduce the accuracy of inferred topologies [74], [76]. We confronted this potential issue by using a constraint tree to leverage strong prior information on deep phylogenetic relationships. In our case, the Angiosperm Phylogeny Group [60] provides a synthesis of well-supported relationships among the plant orders. Overall, bootstrap support for the constrained ML tree was higher than for the unconstrained tree although we had originally expected a stronger effect of using the constraint tree. The fairly high success of recovering recognized orders in the unconstrained analysis likely derives from the large sample size included in this study and the particular genes used; they were chosen, in part, for their high performance in phylogenetic analyses [48], [77].

While this study used a less sparse data matrix than previous work [24], the alignment procedure we use still resulted in a relatively sparse data matrix, particularly for the *trnH-psbA* region. The reason for this is that the SATé alignment algorithm knits together small alignments and introduces gaps when making a consensus alignment [51]. Evidence suggests that introducing gaps does not affect the overall phylogenetic results as seen with the success of phylogenetic reconstructions using super matrix methods that produce extremely sparse alignments [78] and studies that successfully align non-coding *ITS* and chloroplast intergenic spacer data for very large phylogenetic assemblages [79]. These studies suggest that missing data is not critical, particularly if one gene is shared among all taxa. Furthermore, while the effects of missing data on phylogenetic analyses are complex [80], several studies suggest that even taxa with large amounts of missing data can be accurately placed in phylogenies as long as the total number of characters sampled is large (*e.g.,* [81], [82]). In addition, Wiens [80] showed that, in some cases, taxa with large amounts of missing data can improve overall phylogenetic accuracy, particularly with model-based phylogenetic methods (*e.g.*, likelihood) [83]; *but see* [84]. In our case, some instances of non-monophyly of recognized taxonomic groups were caused by individual taxa for which we did not have the full complement of three gene regions. Continued investigation of the influence of missing data on large phylogenetic analyses will help clarify the conditions under which missing data may decrease phylogenetic accuracy.

### Case study: Phylogenetic composition of Puerto Rican forests

Our analysis of Puerto Rican tree communities provides an initial look at broad patterns of phylogenetic structure at a regional scale. For the most part, the ML and Phylomatic phylogenies provided congruent results in terms of NRI, which is a tree-wide metric of phylogenetic composition. In contrast, NTI values, which are more sensitive to variation at the tips of a phylogeny, were not surprisingly, more variable between the two phylogenies. Another difference between the two phylogenies was how the species pool influenced the results. Reducing the scale at which the regional species pool was defined (i.e., from the island to pools in each soil type) caused a decrease for both NRI and NTI when based on the ML phylogeny but no statistically significant change based on the Phylomatic phylogeny.

Based on the island species pool, one of the driest forests (Guánica, which is located at low elevation and on limestone soils) exhibited tree-wide phylogenetic clustering. Across all 15 forests, values of NRI_ISLAND_ tended to decline with mean annual precipitation, suggesting that drier forests generally comprise more phylogenetically clustered subsets of the island species pool than wetter forests. When evaluated with the reduced soil species pool, however, phylogenetic clustering of Guánica became random and only one forest in the moist life zone (Cambalache; located at low elevation and on limestone soils) had significantly clustered NTI with respect to the ML phylogeny only. The two wettest forests (Toro Negro and El Yunque, which are located on higher elevation volcanic soils) exhibited significant phylogenetic evenness in the NRI metric, although the value for Toro Negro was only significant with the ML phylogeny. One interpretation of these patterns is that water limitation represents a strong environmental filter in the dry forests and constrains the composition of local communities to the lineages that are able to persist under these harsh conditions. The issue of water stress in Puerto Rico may be exacerbated by the somewhat confounded nature of underlying geology and precipitation [85]. Specifically, limestone soils tend to occur at lower elevations and receive less precipitation than volcanic soils. The combined influence of these variables likely compounds the effects of limited water availability for plants. In contrast, niche partitioning with respect to other factors (*e.g.*, light use, vulnerability to pathogens) may play a stronger role in the wetter forests on volcanic soils, leading to a phylogenetically more diverse set of co-occurring species. One alternative explanation for this pattern is if *in situ* lineage diversification in Puerto Rico is a more important determinant of local species composition for higher elevation forests. For example, two closely related species of *Tabebuia*, *T. rigida* and *T. schumanniana* (Bignoniaceae) are endemic to El Yunque and Carite mountains, respectively [44].

We acknowledge three main limitations in our ability to interpret these patterns. First, we did not include information on species traits, which are relevant to their occurrence across environmental gradients. Our interpretations depend, in part, on the degree to which functional traits relevant to species occurrence along a gradient of water availability are phylogenetically conserved. Linking key functional traits with phylogenetic relatedness would help to more strongly identify the processes that underlie compositional variation among these forests [70], [86]. Second, the occurrence data we used in this analysis lacks information on species abundances. Our analysis may not detect community assembly processes that are more strongly driven by species relative abundances (i.e., dominance) than the simple presence or absence. Finally, although our null model controls for species richness within each plot, statistical power for detecting nonrandom patterns is low for forests with low species richness [87]. Nonetheless, the observed patterns provide a valuable starting point for future work aimed at addressing these limitations and providing additional insight on tree community variation across broad environmental gradients in Puerto Rico.

We found that values of NRI for each forest based on the different phylogenies were highly correlated whereas NTI values for each forest calculated with the two phylogenies were not correlated. These results reinforce the idea that low resolution among terminal tips (congeneric and confamilial taxa) may be especially problematic for recovering consistent patterns with NTI. In general, previous work has suggested that NRI may have greater power to detect nonrandom patterns of community phylogenetic structure than NTI [2], [88], [89].

In conclusion, our study provides a highly resolved community phylogeny for tropical trees at a regional scale: the island of Puerto Rico. We hope this regional perspective facilitates additional work to better understand the processes governing composition of local tree communities. Our case study confirms the value of a highly resolved phylogeny for detecting nonrandom patterns of phylogenetic community composition. Together with the extensive amount of existing data available in Puerto Rico on environmental conditions, land use history, species distributions and functional traits, we anticipate that the regional phylogeny provided here will help strengthen our historical perspective on the forces generating and structuring the diversity of Puerto Rican forests.

## Supporting Information

Table S1 **Table of molecular sequences from Puerto Rican tree species included in this analysis, including taxonomic information, collection details, GenBank accessions and voucher specimen details.**
(XLSX)

Appendix S1 **Input file for PATHd8 [61] used to date the constrained ML tree.**
(TXT)

Appendix S2 **Input file for PATHd8 [61] used to date the unconstrained ML tree.**
(TXT)

Appendix S3 **A Newick format file (.new) containing five phylogenies for Puerto Rican trees: (1) the dated ultrametric 50% consensus ML phylogeny using ordinal-level (APG-III) constraints, (2) the dated ultrametric 50% consensus ML phylogeny without topological constraints, (3) the undated non-ultrametric 50% consensus ML phylogeny using ordinal-level (APG-III) constraints, (4) the undated non-ultrametric 50% consensus ML phylogeny without topological constraints, (5) a corresponding Phylomatic phylogeny.** Bootstrap support is provided for ML trees (i.e., trees 1–4; NA's in trees 1 and 3 refer to APG-III constrained nodes).
(NEW)

Appendix S4 **Detailed results from ‘nodesig’ analysis [67] for each forest using the dated and constrained 50% consensus ML phylogeny and Phylomatic phylogeny.**
(PDF)
