# Supplementary material for: A Well-Resolved Phylogeny of the Trees of Puerto Rico Based on DNA Barcode Sequence Data
Source: PLoS One. 2014 Nov 11;9(11):e112843. doi: 10.1371/journal.pone.0112843 (PMC4227909; doi:10.1371/journal.pone.0112843)

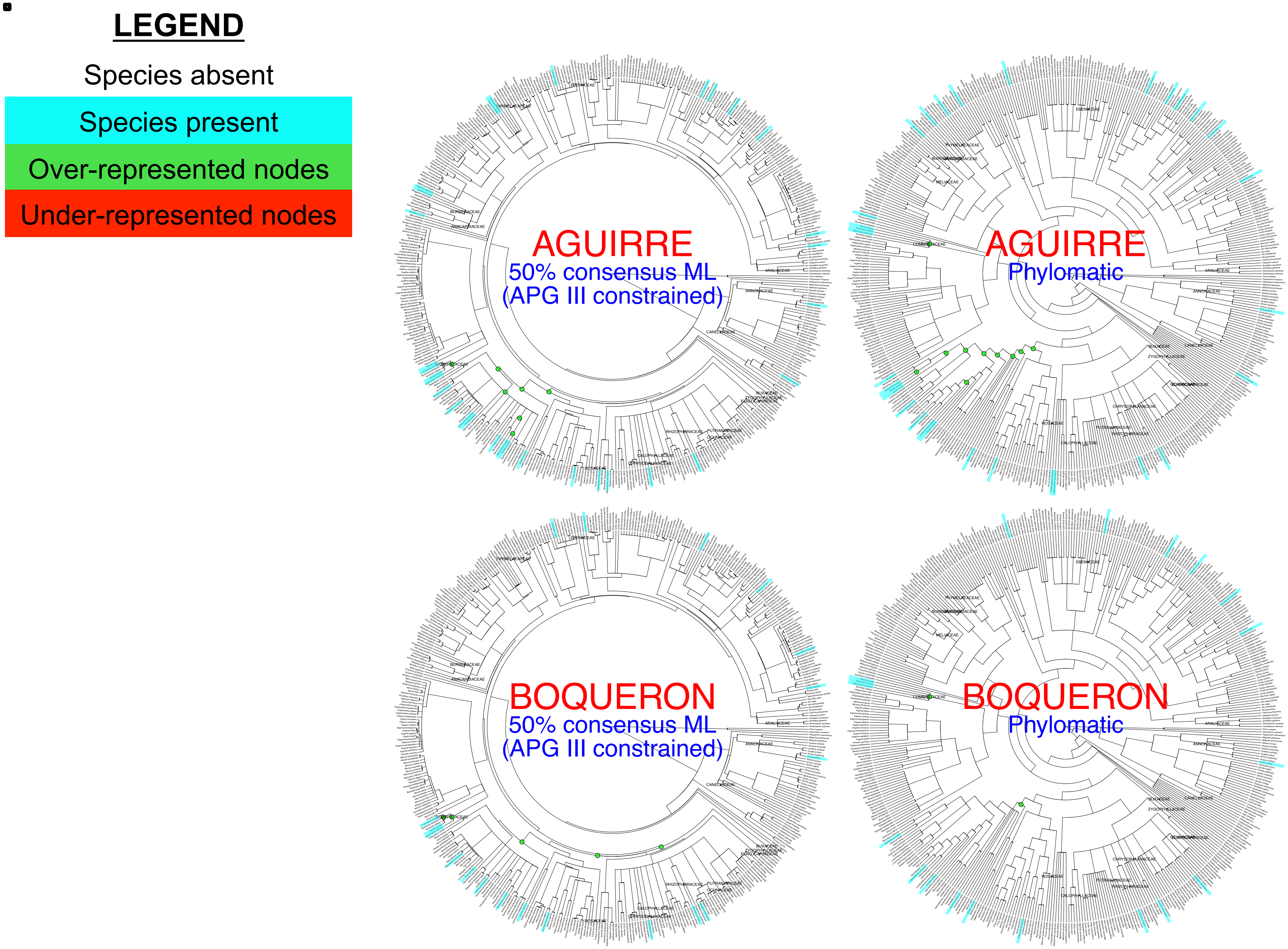
LEGEND
Species absent
Species present
Over-represented nodes
Under-represented nodes
AGUIRRE
50% consensus ML
(APG III constrained)
AGUIRRE
Phylomatic
BOQUERON
50% consensus ML
(APG III constrained)
BOQUERON
Phylomatic

LEGEND

Species absent

Species present

Over-represented nodes

Under-represented nodes

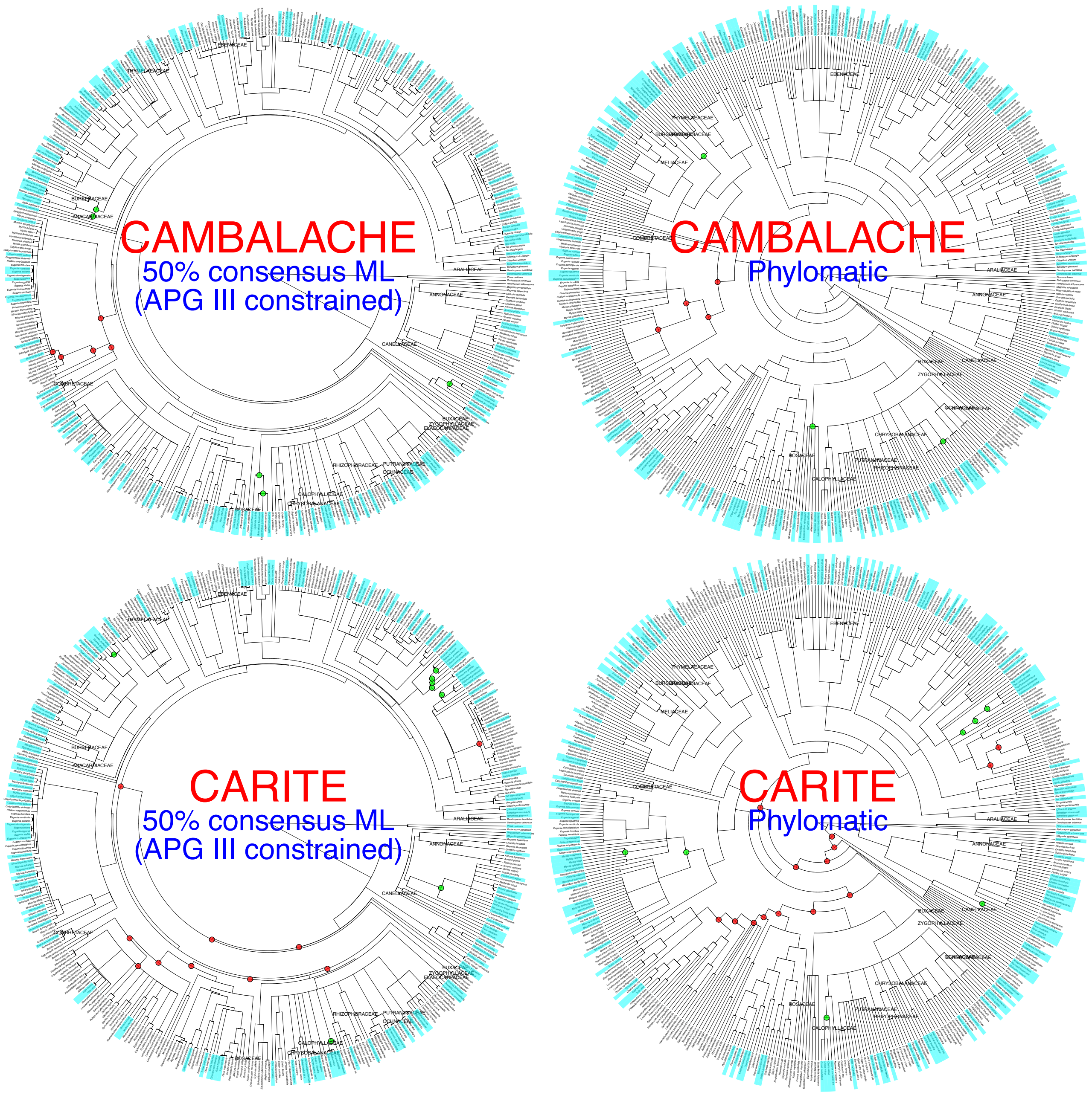

LEGEND

Species absent

Species present

Over-represented nodes

Under-represented nodes

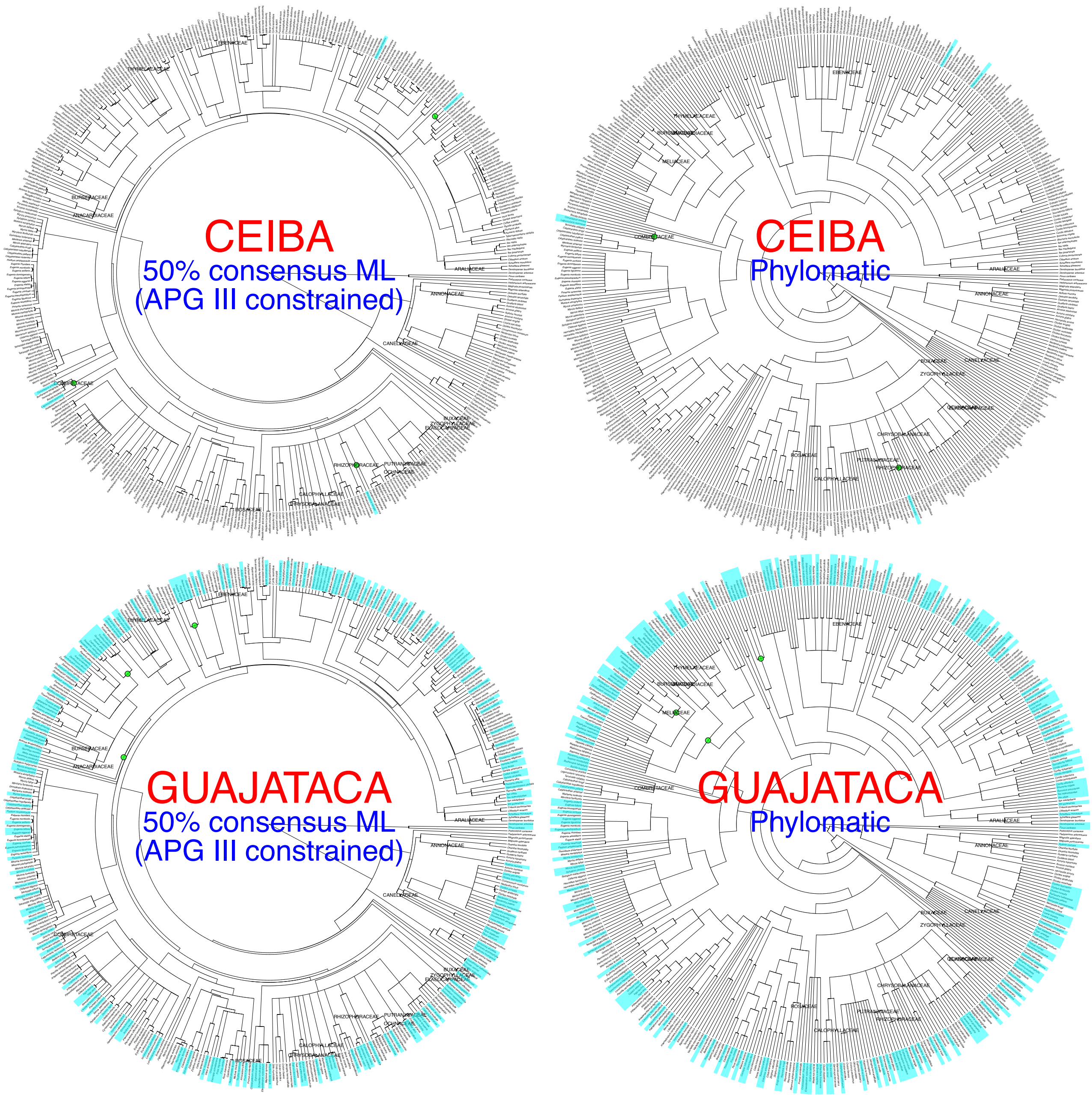

LEGEND

Species absent

Species present

Over-represented nodes

Under-represented nodes

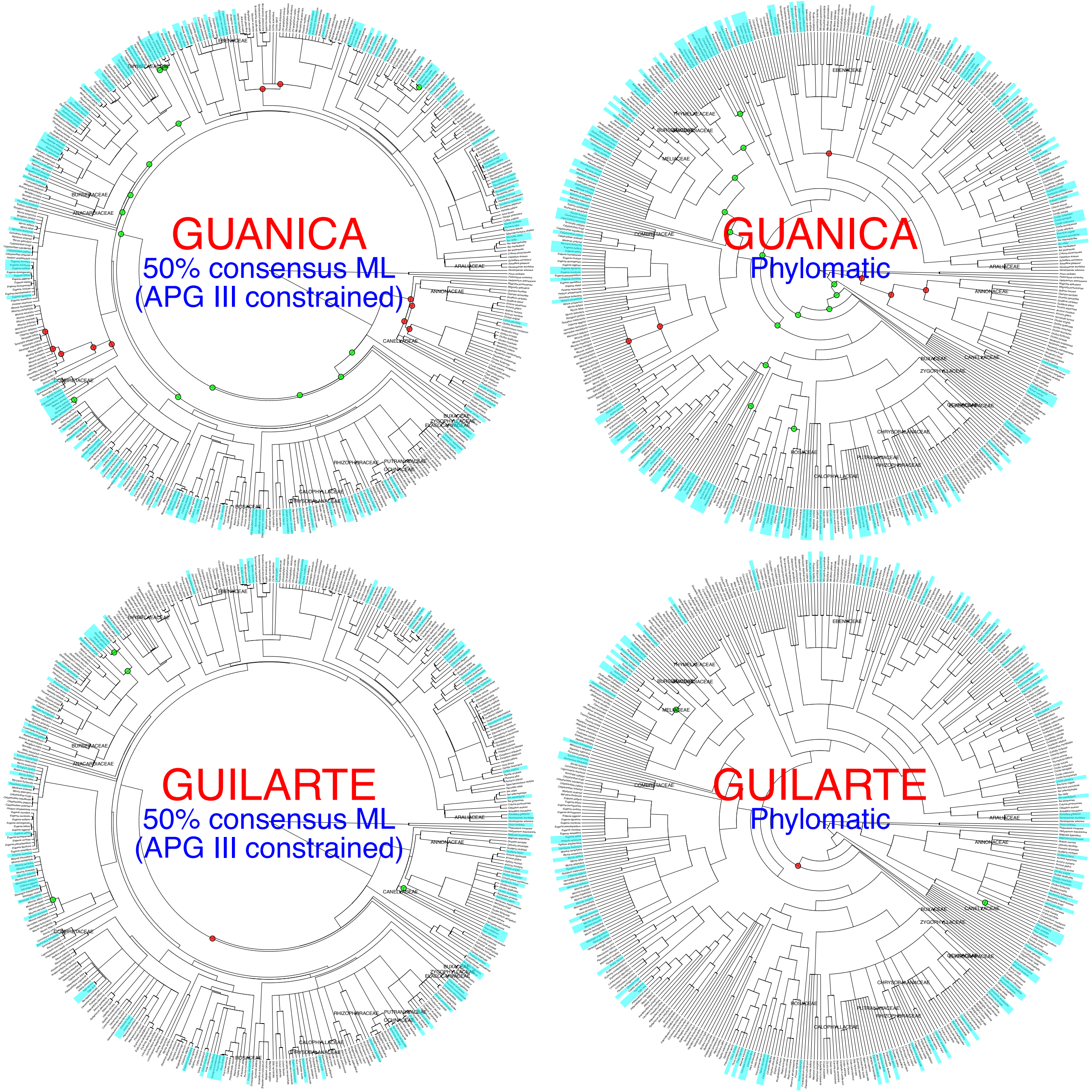

LEGEND

Species absent

Species present

Over-represented nodes

Under-represented nodes

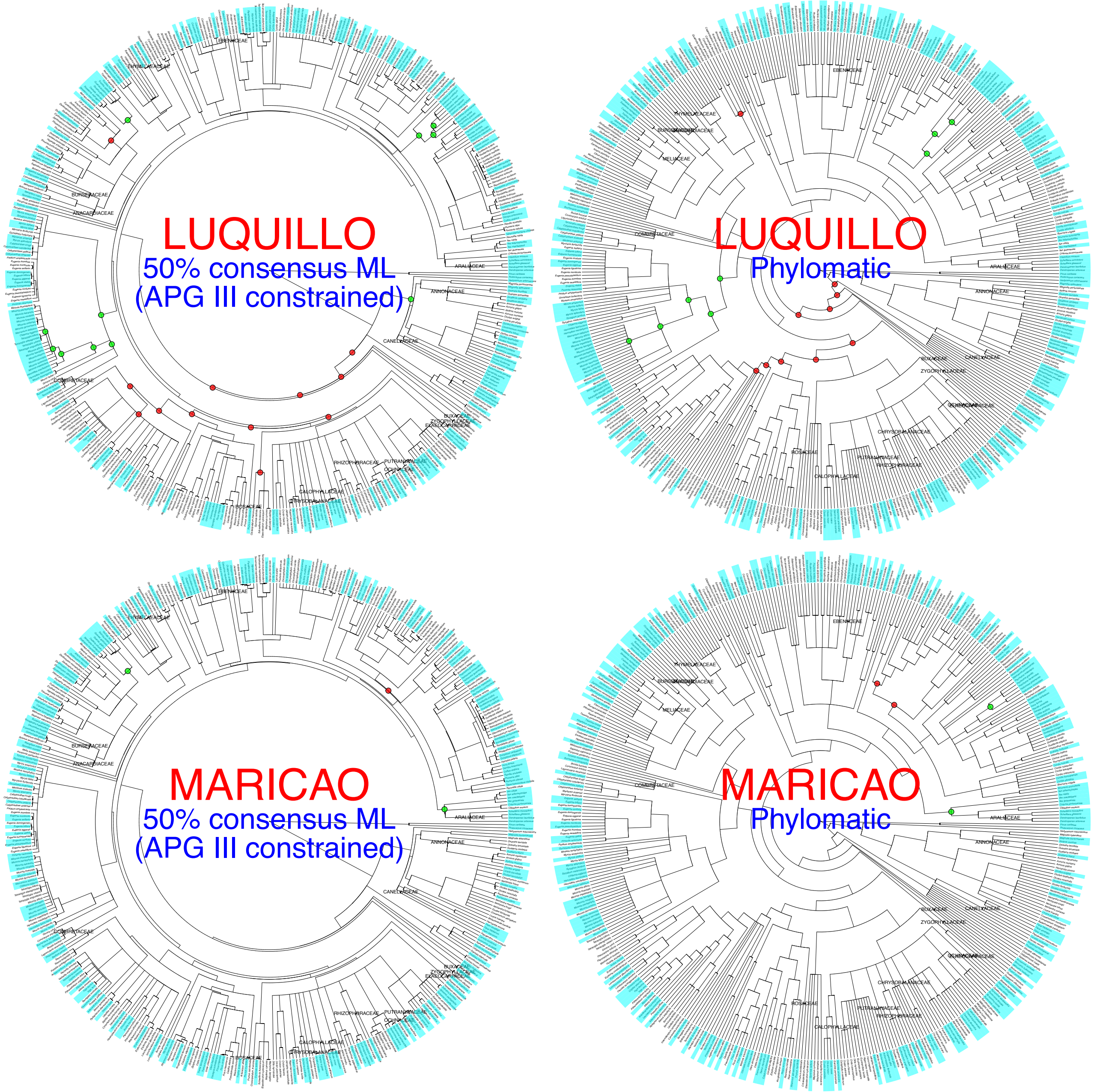

LEGEND

Species absent

Species present

Over-represented nodes

Under-represented nodes

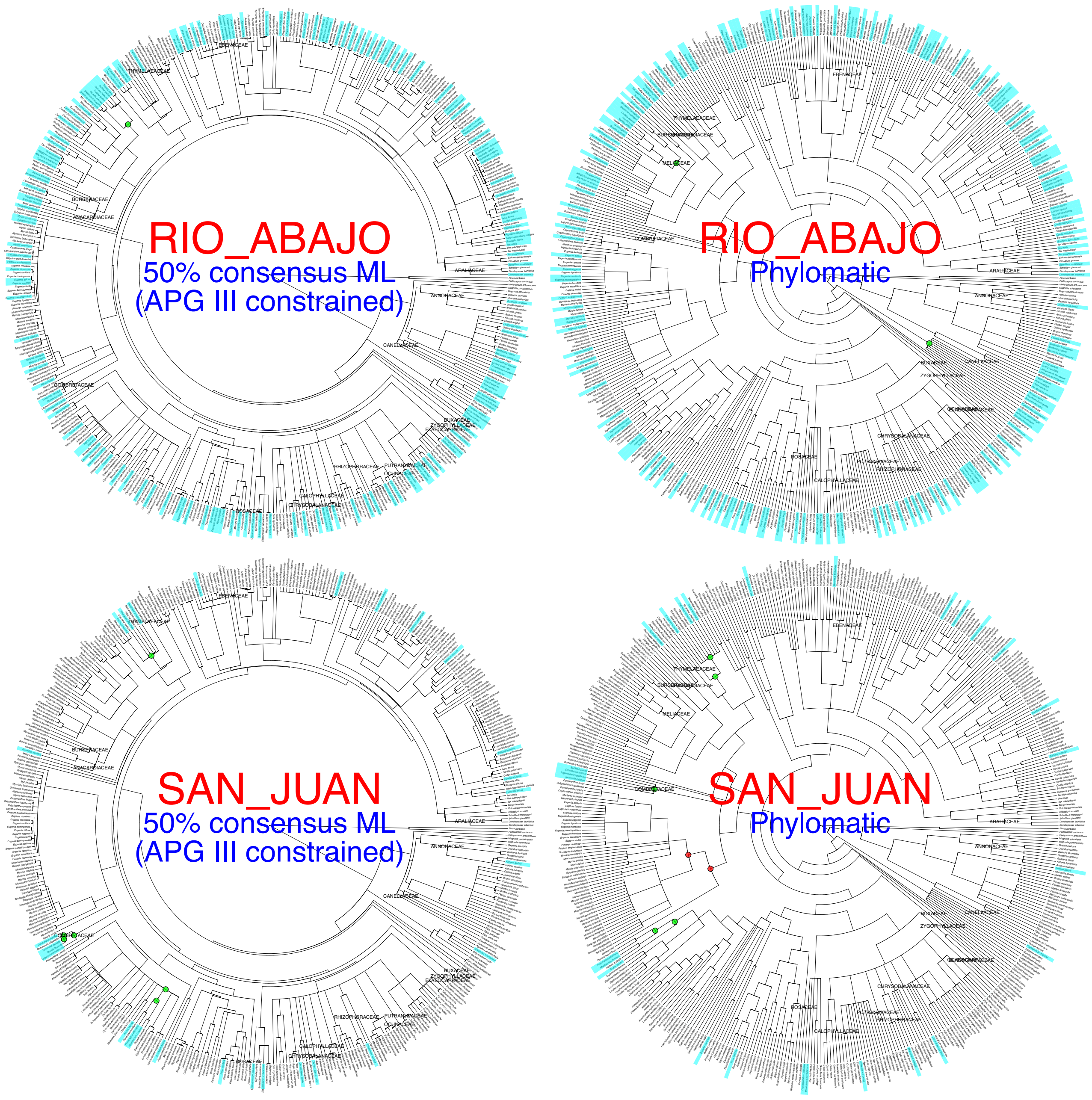

LEGEND

Species absent

Species present

Over-represented nodes

Under-represented nodes

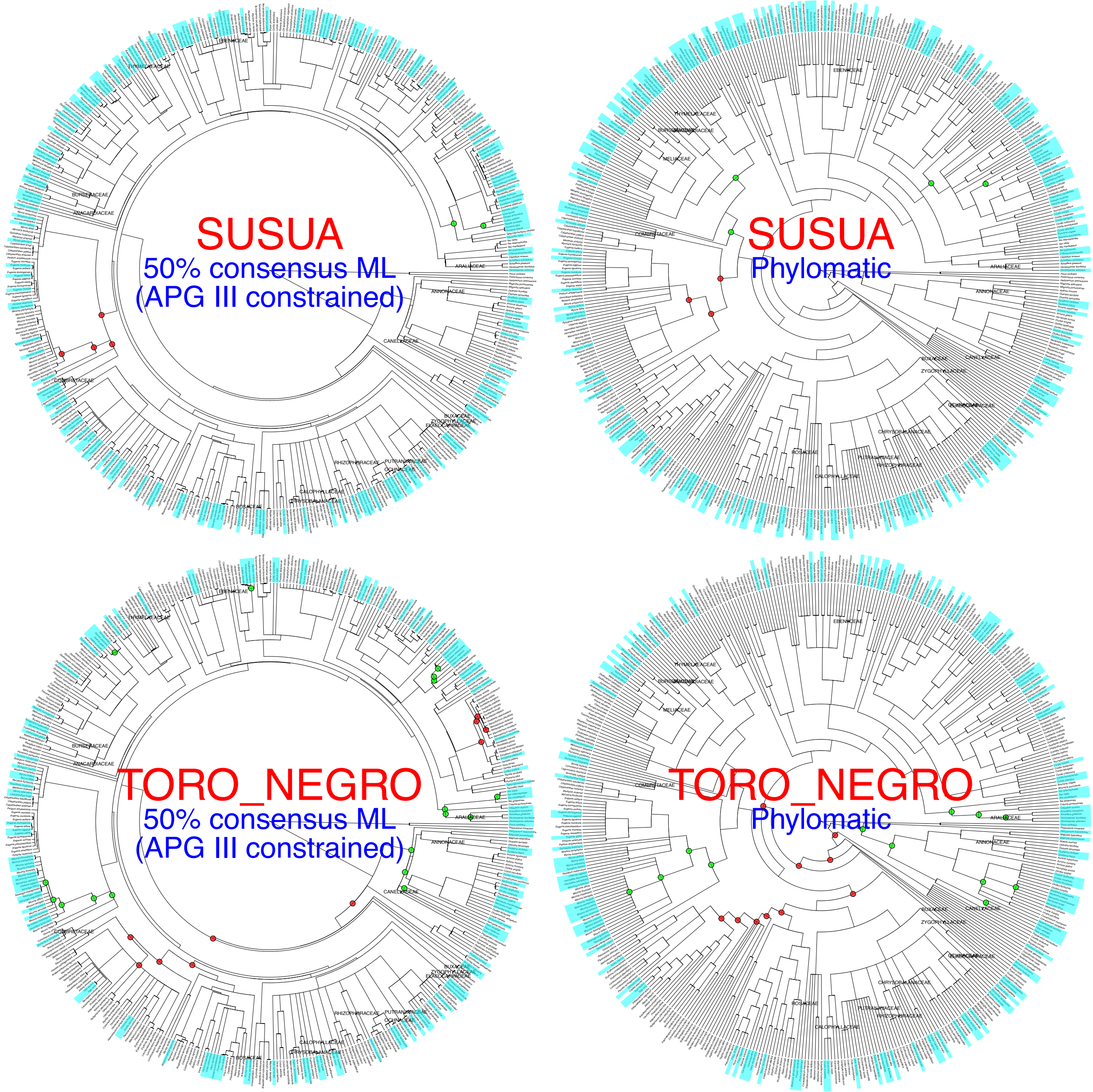

LEGEND

Species absent

Species present

Over-represented nodes

Under-represented nodes

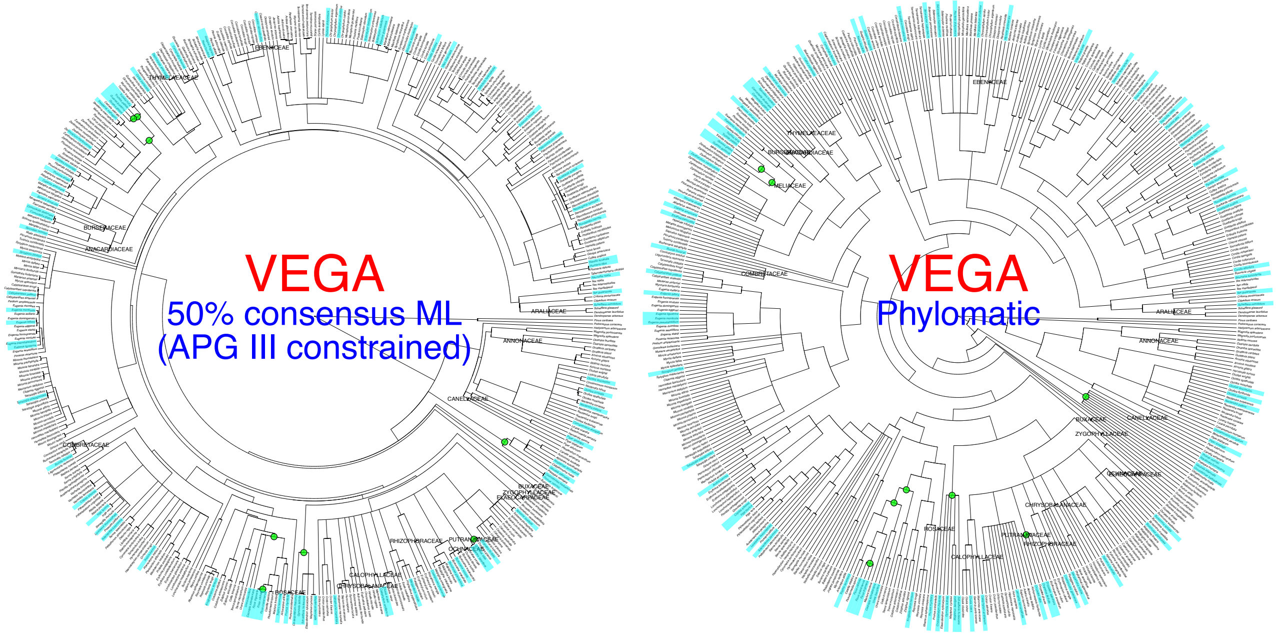

Supplement: Appendix S4 — Detailed results from ‘nodesig’ analysis [67] for each forest using the dated and constrained 50% consensus ML phylogeny and Phylomatic phylogeny. (PDF) [file pone.0112843.s005.pdf]
